# Excitatory and Inhibitory Synaptic Imbalance Caused by Brain-Derived Neurotrophic Factor Deficits During Development in a Valproic Acid Mouse Model of Autism

**DOI:** 10.3389/fnmol.2022.860275

**Published:** 2022-04-06

**Authors:** Chuchu Qi, Andi Chen, Honghui Mao, Erling Hu, Junye Ge, Guaiguai Ma, Keke Ren, Qian Xue, Wenting Wang, Shengxi Wu

**Affiliations:** ^1^Department of Neurobiology, School of Basic Medicine, Fourth Military Medical University, Xi’an, China; ^2^MOE Key Laboratory of Modern Teaching Technology, Center for Teacher Professional Ability Development, Shaanxi Normal University, Xi’an, China; ^3^Department of Physiology, Medical College of Yan’an University, Yan’an, China

**Keywords:** synaptic development, E/I balance, autism, BDNF, excitatory synapse, inhibitory synapse, valproic acid

## Abstract

Environmental factors, such as medication during pregnancy, are one of the major causes of autism spectrum disorder (ASD). Valproic acid (VPA) intake during pregnancy has been reported to dramatically elevate autism risk in offspring. Recently, researchers have proposed that VPA exposure could induce excitatory or inhibitory synaptic dysfunction. However, it remains to be determined whether and how alterations in the excitatory/inhibitory (E/I) balance contribute to VPA-induced ASD in a mouse model. In the present study, we explored changes in the E/I balance during different developmental periods in a VPA mouse model. We found that typical markers of pre- and postsynaptic excitatory and inhibitory function involved in E/I balance markedly decreased during development, reflecting difficulties in the development of synaptic plasticity in VPA-exposed mice. The expression of brain-derived neurotrophic factor (BDNF), a neurotrophin that promotes the formation and maturation of glutamatergic and GABAergic synapses during postnatal development, was severely reduced in the VPA-exposed group. Treatment with exogenous BDNF during the critical E/I imbalance period rescued synaptic functions and autism-like behaviors, such as social defects. With these results, we experimentally showed that social dysfunction in the VPA mouse model of autism might be caused by E/I imbalance stemming from BDNF deficits during the developmental stage.

## Introduction

Autism spectrum disorder (ASD) is a complex developmental neurological disorder that is characterized by social interaction deficits, restricted interests and repetitive behaviors. ASD is diagnosed 1 in 44 children according to the latest data from the Centers for Disease Control and Prevention (CDC) of the United States. The incidence of autism is affected by many factors. Medication during pregnancy is one of the common factors (Chaste and Leboyer, 2012; Varghese et al., 2017). Valproic acid (VPA), an anticonvulsant or anti-seizure drug, has been reported to dramatically elevate ASD risk in offspring taken during in pregnancy (Brumback et al., 2018). VPA exposure of the embryos of pregnant rodents replicates ASD-like symptoms, including social deficits and repetitive behaviors, and this damage has been found to occur at an early developmental stage, affecting neurodevelopment (Wang et al., 2018).

Development is a crucial and sensitive period that governs the temporal and spatial profile of excitatory and inhibitory synapses in the developing brain (Ismail et al., 2017). Excitatory inputs come from glutamatergic neurons, while inhibitory inputs are mainly regulated by GABAergic neurons. During the neurodevelopmental stage, excitatory and inhibitory inputs do not work independently but in a tight balance (the E/I balance), which is important for brain function (Lee et al., 2017). Accordingly, disruption of the E/I balance at the synaptic level has been implicated in various mental disorders, including schizophrenia, epilepsy and ASD. Importantly, previous studies have reported that the balance of neuronal excitation and inhibition is altered in the brain of a VPA-induced ASD rodent model (Gogolla et al., 2009; Lee et al., 2014; Lenart et al., 2020). However, these studies have generally concentrated on either changes in excitation or disruptions in inhibition. In contrast, the neuronal E/I balance involves regulation at synaptic levels in local circuits (Lee et al., 2017), and the excitatory and inhibitory components regulate and restrict each other. Therefore, to explore the consequences of this imbalance, it seems important to study these two parts as a system.

At all stages of brain development, neurotrophins play a pivotal role in generating precisely connected networks at the level of synapses (Niculescu et al., 2018). One growth factor that has a compelling function in neuronal development is brain-derived neurotrophic factor (BDNF), which participates in axonal and dendritic differentiation during embryonic stages, as well as in the formation and maturation of glutamatergic and GABAergic synapses during postnatal development (Chapleau et al., 2009; Gottmann et al., 2009). BDNF can play a permissive role in shaping synaptic networks, making them more susceptible to plasticity. Previous articles have reported that BDNF is related to the occurrence and development of autism. Most of these studies have reported alterations in neurotrophic factor levels in individuals with ASD; signaling pathways concerning BDNF are involved in regulating dendrite differentiation in ASD (Reim and Schmeisser, 2017; Ka and Kim, 2018). However, whether BDNF affects the E/I balance at the synaptic level remains unclear.

The anterior cingulate cortex (ACC) is a part of the agranular frontal cortex, which belongs to the limbic system. This area is involved in disparate cognitive and emotional processes, such as frontal executive functions, parietal sensorimotor systems, and intentional processes (Burgos-Robles et al., 2019). Recently, it was reported that when human participants engage in social interaction, the ACC is markedly activated (Rudebeck et al., 2006). Studies have begun to investigate the role of ACC region in social decision-making since the ACC is functionally connected to a broad set of regions engaged in social information processing (Chang et al., 2013; Apps et al., 2016). Because of the extraordinary significance of social behavior in mammals and the relationship between social behavior and the ACC, abnormalities in the structure or function of the ACC affect natural and necessary social behaviors. Previous research has reported that the ACC is involved in sociability and aggression in BALB/cJ mice (van Heukelum et al., 2019). Our previous study revealed that dysfunction of pyramidal neurons in the ACC induces social deficits in a *Shank3*-deficient ASD mouse model (Guo et al., 2019). However, developmental changes in the E/I balance in the ACC of an ASD mouse model remain poorly understood.

Therefore, our study aimed to determine a critical period of E/I balance in the ACC, when we could regulate synaptic developmental plasticity to rescue autism-like behaviors. In the current study, we confirmed that VPA exposure in pregnancy induced autistic-like phenotypes in mice, including decreases in USVs, social deficits, and repetitive behavior. We found that the influence of VPA increased over time, resulting in an E/I imbalance. In early developmental stages, there were deficits in excitatory input, and in later stages, both excitatory and inhibitory input dysfunction occurred, as well as E/I imbalance. BDNF expression might play a crucial role in this change, as it concurrently modulates excitatory and inhibitory synapses. In terms of the prevalence of ASD, it is imperative to investigate the progression of this disorder during development, as findings could be utilized for preventive therapies and facilitate understanding of the mechanisms of neurodevelopmental disorders.

## Materials and Methods

### Animals and Housing

Adult male and female C57BL/6J mice weighing 20–25 g were housed in a vivarium under environmentally controlled conditions [24 ± 2°C; night-day cycle of 12/12 h (lights on at 08:00)] with *ad libitum* access to food and water. Female mice were housed in groups of three per cage, and male mice were housed in pairs. All experimental procedures were approved by the Institutional Animal Care and Use Committee (IACUC) of the Fourth Military Medical University (Approval No. IACUC-20200528) and carried out according to the “Principles of Medical Laboratory Animal Care” issued by the National Ministry of Health in China. All efforts were made to minimize animal suffering and to reduce the number of animals.

### The Valproic Acid Mouse Model

Following the methods of previous reports (Luhach et al., 2021), pregnant female dams received a single injection of VPA (Sigma-Aldrich, St. Louis, MO, United States) at a dose of 500 mg/kg (i.p.) dissolved in 0.9% w/v sterile saline on gestational day 12.5; the saline groups received isopycnic saline at the same time. Before parturition, pregnant dams were housed individually. Pups were weaned at P21 and separated according to sex, with 3–4 mice per cage. Male and female mice were used for testing prior to weaning, but only male mice were used for behavioral tests beyond that time point, given the higher susceptibility of males to ASD in the VPA model (Kataoka et al., 2013). All behavioral experiments were performed at 24 ± 1°C.

### Behavioral Assessment

#### Ultrasonic Vocalizations

Neonatal P7 and P14 mice were placed in a square transparent container (32 cm × 20 cm × 30 cm) with an ultrasonic microphone inserted through the open top. Sounds were recorded with Ultramic384K BLE (Dodotronic, France) ultrasound microphones that feature a flat frequency response from 0 to 192 kHz. The sampling rate was 384 kHz, and data were recorded in a 16-bit format. Data acquisition (SeaWave 2.0; Avisoft Bioacoustics) and assessment software (Deep Squeak 3.0)^1^ were used to record and analyze the calls. We analyzed the following three aspects: (a) the number of USVs from 25 to 90 kHz; (b) the call length (duration of the call, measured in milliseconds), and (c) the principal frequency.

#### Sociability

At age P30, male mice were individually tested for sociability and social preference using three-chamber apparatus. Mice were placed in the test room to habituate for 1–2 h. The apparatus was made of black perspex and consisted of three identical chambers (30 cm × 45 cm × 20 cm), with the central compartment containing two sliding doors (6 cm × 20 cm) that opened into the adjacent chambers. Two cylindrical grid cages were placed in the corners of the outer chambers, allowing free interaction (visual, olfactory, and auditory) between the subject mouse and caged mouse. The top of the cages was capped with a cone roof. The test session began with the subject mouse habituating for 10 min in the central chamber. After an empty cage and a target mouse were positioned in the cylindrical cages, the subject mouse was then allowed access to all three chambers during a 10 min recorded trial. In the social preference test, another stimulus mouse was added to the empty cage, and then the subject mouse was allowed access to all three chambers during a 10 min recorded trial. The video recordings were analyzed and the time spent in each chamber, particularly in proximity to the cage, was recorded. Time in each zone was measured by an automated analysis system SMART 3.0 (Panlab S.L.U., Spain). The apparatuses were cleaned with 75% alcohol after each test. To measure sociability, the preference score was calculated as follows: time spent with social zone minus time spent with empty zone/total time spent with social zone and empty zone. To assess social novelty, we calculated a preference score for each mouse as follows: time spent with stranger mouse minus time spent with original mouse/total time spent with stranger mouse and original mouse. This calculation method is a reliable and proven way to analyze the discrepancy between two groups (Scheggia et al., 2020).

#### Grooming

The grooming test was performed at P30. The grooming apparatus was an acrylic cage (20 cm × 20 cm × 25 cm) with three opaque walls, one clear wall, and an opaque bottom. The recording started as soon as the mouse was put into the cage and lasted 30 min. After each test, the chamber was cleaned with 75% alcohol solution. The number of grooming sessions, total grooming time and latency time of the first session were manually marked on a computer screen and counted by an experienced researcher blind to the treatment.

#### Elevated Plus Maze

An elevated plus maze (EPM) test was performed at P32. The EPM apparatus consisted of two open arms (50 × 10 cm), two closed arms of the same size with 40 cm high walls, and a center platform (10 × 10 cm). The apparatus was elevated to a height of 50 cm above the test room floor. Mice were placed in the central area facing one of the open arms at the start of the test. Behaviors were observed for 5 min, and the travel trace was measured by SMART 3.0. The apparatus was cleaned with 75% alcohol after each test.

#### Marbles Burying Test

The marbles burying test (MBT) was conducted after the EPM and carried out as previously described (Qi et al., 2018). First, the cage was prepared with wood chip bedding that was lightly tamped down to create a flat surface. Mice were provided with a habituation session and then 12 regular glass marbles were placed on the surface, each approximately 4 cm apart. Then, the mouse was placed in the cage, and recorded for 20 min. When the test was finished, the video analyzed to determine the number of marbles buried.

#### Open Field Test

The open field test (OFT) was performed after MBT. The test was carried out as previously described (Qi et al., 2018). Mice were placed in the center of a cubic chamber (40 cm × 40 cm × 40 cm). Each trial was recorded for 10 min and time in the center and outer zone and the numbers of entries were measured by an automated analysis system (SMART 3.0). After each test, the chamber was cleaned with 75% alcohol solution.

### Immunofluorescence Staining

For the immunofluorescence studies, the mice were deeply anesthetized with isoflurane and then transcardially perfused with 0.01 M phosphate-buffered saline (PBS; pH 7.4), followed by 5, 10, or 30 mL of 4% paraformaldehyde (PFA) at P7, P14, or P30, respectively. The brains were removed and fixed in 4% PFA for 2 h and then put into 20 or 30% sucrose at 4°C for 72 h. The whole brain was cut into 30- or 40-μm sections on a cryotome (Leica, Germany). The sections were incubated at room temperature overnight with a mixture of anti-VGLUT1 guinea pig antibody (1:200, Synaptic Systems), anti-PSD95 rabbit antibody (1:300, Abcam), anti-VGAT rabbit antibody (1:500, Synaptic Systems), anti-TUJ1 mouse antibody (1:200, Abcam) or anti-gephyrin mouse antibody (1:200, Synaptic Systems) and anti-MAP2 rabbit antibody (1:100, Abcam) in 0.01 M PBS containing 0.3% Triton X-100, 0.3% κ-carrageenan, and 1% donkey serum. The sections were further incubated for 3 h at room temperature with a mixture of Alexa 488- or Alexa 594-conjugated donkey anti-mouse, rabbit or guinea pig IgG (1:800, Invitrogen). Images were captured using a laser scanning confocal microscope (FV3000, Olympus, Japan). The number and fluorescence intensity of VGLUT1-, PSD95-, VGAT-, and gephyrin-positive terminals were measured by using Image-Pro Plus (Version 6.0). Briefly, we selected the target cell based on their similar nucleus and skelemin morphology with blinding the group information. The target cells were circled by 20 μm diameter. Then the “area” and “IOD” in measurements were selected. Next, we adjusted the brightness range to exclude the background and make all positive puncta be covered and be counted. The “count number” is the “number of puncta” and “IOD(sum)/area(sum)” serves as “a.u. (Arbitrary Unit)” to describe fluorescence intensity.

### Western Blot

After the behavioral tests, on P7, P14, or P30, mice were anesthetized with isoflurane, ACC tissues were separated and quickly put into RIPA buffer (BiYunTian, China) consisting of protease and phosphatase inhibitors (Roche, CH). The protein concentration of all of the samples was 2 μg/μL, measured using the BCA Protein Assay Kit (Thermo, United States). The process has been previously described (Qi et al., 2018). The membranes were incubated with horseradish peroxidase-conjugated anti-rabbit or mouse IgG antibody (1:1000, Cell Signaling Technology) for 1 h at room temperature. The primary antibodies were as follows: VGLUT1 (1:1000, Millipore), PSD95 (1:1000, Abcam), VGAT (1:1000, Synaptic Systems), gephyrin (1:1000, Synaptic Systems), BDNF (1:1000, Abcam), GAPDH (1:1000, Proteintech) and β-actin (1:1000, Proteintech). The images were scanned by a chemiluminescent imaging system (5200 Multi, Tanon, China or UVP ChemStudio PLUS, Analytik Jena, Germany) and were quantified with ImageJ software (Version 1.48). GAPDH and β-actin served as the staining standard.

### *In vitro* Electrophysiology Recording

The slice preparations were performed as previously described (Guo et al., 2019). Briefly, mice were anesthetized with pentobarbital sodium (30 mg/kg body weight) and transcardially perfused with ice-cold carbogenated (95% O_2_, 5% CO_2_) cutting solution containing 115 mM choline chloride, 2.5 mM KCl, 1.25 mM NaH_2_PO_4_, 0.5 mM CaCl_2_, 8 mM MgCl_2_, 26 mM NaHCO_3_, 10 mM D-(+)-glucose, 0.1 mM L-ascorbic acid, and 0.4 mM sodium pyruvate (with osmolarity of 295–300 mOsm/L). The brains were then rapidly removed and placed in an ice-cold cutting solution for slice preparation. Coronal slices (300 μm) containing the ACC were prepared by a slicer (VT1200 S, Leica, Germany) and then incubated in a holding chamber at 32°C with a carbogenated cutting solution for 30 min. The slices were then transferred to artificial cerebral spinal fluid containing 119 mM NaCl, 2.3 mM KCl, 1.0 mM NaH_2_PO_4_, 26 mM NaHCO_3_, 11 mM D-(+)-glucose, 1.3 mM MgCl_2_, and 2.5 mM CaCl_2_ (pH 7.4, with osmolarity of 295–300 mOsm/L) at room temperature for 1 h. Recording pipettes were filled with a solution as previously described (Guo et al., 2019). The cell membrane potential was clamped at −65 mV and +10 mV in the presence of 1 μM tetrodotoxin (Taizhou Kang Te, China) to record miniature excitatory postsynaptic currents (mEPSCs) and miniature inhibitory postsynaptic currents (mIPSCs), respectively. For the mEPSCs and mIPSCs results, 5 min recordings were performed in the gap-free voltage-clamp mode. The last 3 min of mEPSCs and mIPSCs events were automatically detected and manually re-checked with Mini Analysis (6.0.3, Synaptosoft, United States).

### Primary Cell Culture

#### Isolation and Culture of Primary Cortical Neurons

Primary cortical neurons were prepared from the brains of 16-day-old C57BL/6J mouse fetuses. The pregnant mouse was sacrificed, and the fetus was taken out of the uterus and placed in ice-cold Hank’s balanced salt solution (HBSS, Sigma). The fetus’s brain was removed, and the cortex was dissected and collected. After collecting all cortices, the cortical pieces were triturated with a Pasteur pipette and allowed to settle for 5 min before the supernatant was transferred to a new 15 mL tube. This procedure was repeated three times. The cell suspension obtained was centrifuged at 300 × *g* for 5 min. Cell pellets were resuspended in neurobasal medium (Gibco, United States) supplemented with 2% B27 (Gibco, United States), 2 mM glutamine (Sigma, United States), 100 U/mL penicillin, and 100 μg/mL streptomycin and seeded onto poly-l-lysine-coated 35-mm dishes or coverslips. Half of the medium was changed every 3 days.

#### Transfection With siRNAs

The siRNA used to silence mouse BDNF (forward: 5′-GGACGGUCACAGUCCUAGATT-3′, reverse: 5′-UCUAGGACUGUGACCGUCCTT-3′) and a negative siRNA control were obtained from Tsingke Biotechnology Company, China. For *in vitro* BDNF knockdown, the primary cortical neurons were transfected with siRNA against BDNF or the negative control siRNA using Lipofectamine RNAiMAX reagent (Invitrogen Life Science, United States) according to the recommendations of the manufacturer. Three days later, cells were harvested for qRT-PCR or immunocytochemistry assays as indicated. We also observed development of glutamatergic pyramidal neurons and GABAergic interneurons under this transfection after 12 DIV by immunocytochemistry assays. Total ribonucleic acid (RNA) was extracted with TRIzol reagent, and PrimeScript™ RT Master Mix (TaKaRa, Japan) was used to reverse transcribe the total RNA (500 ng) into complementary DNA. Real-time PCR was carried out using a StepOnePlus™ Real-Time PCR Instrument (Thermo, United States) with 2X Universal SYBR Green Fast qPCR Mix (ABclonal, United States). The sequences of the primers used were as follows:

BDNF: Forward: 5′-GCCCATTTCCCTAGTCAG-3′,
Reverse: 5′-TGAAGCGATTGTTTGCC-3′;
GAPDH: Forward: 5′-CAAAATGGTGAAGGTCGGTG TG-3′,
Reverse: 5′-TGATGTTAGTGGGGTCTCGCTC-3′.

#### Neurotrophin Treatment

Primary cortical neurons were allowed to establish for 3 days, after which 500 ng/mL recombinant human BDNF (PeproTech, United States) was added, and then the cultures were kept for the next 3 days *in vitro*.

#### Immunocytochemistry

For the immunocytochemistry assay, primary cortical neurons were fixed in 4% paraformaldehyde for 15 min at room temperature followed by incubation with 0.25% Triton X-100 for 10 min. Fixed cells were then incubated with primary antibodies including NeuN (1:500, Millipore), CaMKII (1:200, Abcam), MAP2 (1:100, Abcam), and GABA (1:200, GeneTex) overnight at 4°C as indicated, followed by incubation with the secondary fluorescent antibody for 1 h at room temperature. Finally, the samples were incubated with DAPI for nuclear staining and visualized with an FV3000 confocal microscope (FV3000, Olympus, Japan).

### Pharmacological Rescue Experiments

For the BDNF treatment experiments, mice were randomly separated into two groups: one that was injected with 114 μl BDNF (0.0013 μg/μl, PeproTech, United States) into ACC for 3 days in succession and one that was injected with isovolumetric saline. For the BDNF infusion to the ACC, a unilateral stainless steel guide cannula (ID 0.14 mm, OD 0.3 mm, BIC-3, RWD Life Science, China) was implanted into the right ACC according to the following coordinates (anteroposterior: +0.73; dorsoventral: −1.75; mediolateral: −0.28) and secured to the skull with dental cement. Then, a mini osmotic pump (1003D, RWD Life Science, China) was embedded into subcutaneous tissue connected to the cannula, releasing the drug to the ACC at a 1.0 μl/hr for 3 days. After treatment, we performed behavioral assessments, electrophysiological tests and Western blot analyses.

### Statistical Analysis

All data are presented as the means and standard error of the mean. All statistical analyses and graph plotting were performed using GraphPad Prism 8.0 (GraphPad Software, Inc., United States). Statistical comparison of two means was performed by unpaired two-tailed Student’s *t*-test or paired two-tailed Student’s *t*-test, while multiple comparisons were performed using one-way ANOVA. n indicates the number of animals tested, unless otherwise indicated. *P* < 0.05 was considered significant.

## Results

### The Valproic Acid Mouse Model Showed Autistic-Like Behaviors Throughout Development

**FIGURE 1:**
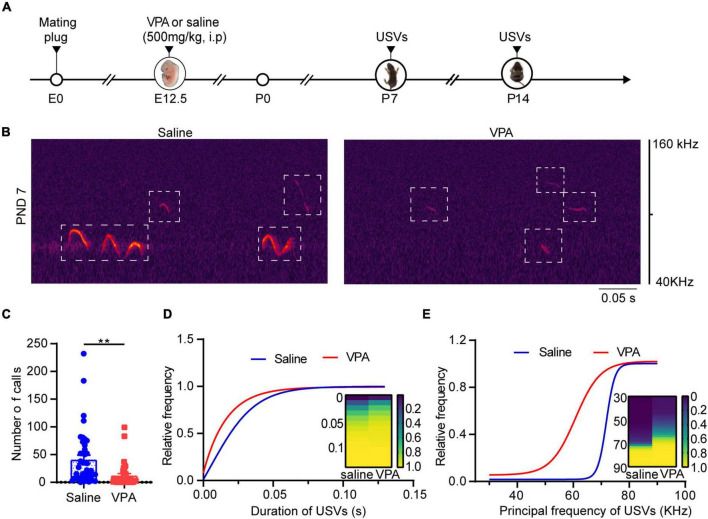
VPA-exposed pups expressed decreasing calls of attention at P7. **(A)** Timeline of VPA-exposed autism model making and USVs recording. USV, ultrasonic vocalization. **(B)** Left: Canonical waveforms of USVs from the saline group. Right: Canonical waveforms of USVs from the VPA group. Data are presented **(C)** number of calls, **(D)** duration, **(E)** principal frequency of the calls (Saline: *n* = 42; VPA: *n* = 40). Data are presented as the mean ± SEM. **p < 0.01 vs. saline-exposed group by two-tailed unpaired Student’s *t*-test.

Ultrasonic vocalizations are a socially relevant form of communication in rodents during the perinatal and neonatal periods, and mice share some similarities with humans in terms of the anatomical structure used to produce vocalizations (Arriaga et al., 2012). It has been reported that VPA intake reduces the frequency and number of USVs in pups (Moldrich et al., 2013); we investigated whether this change also occurs at different developmental stages. We observed that at P7 and P14, pups produced ultrasonic vocalizations after being separated from their mothers in both the saline and VPA groups (Figures 1B–E, *n* = 42 of saline group, *n* = 40 of VPA group; Supplementary Figures 1A–D, *n* = 11 of saline group, *n* = 9 of VPA group). Our results showed that the number, duration, and principal frequency of calls were lower in the VPA group than the saline group. At P7, the number (Figure 1C, *P* = 0.0011), duration (Figure 1D) and principal frequency (Figure 1E) of calls in the VPA group differed, while at P14, the number (Supplementary Figure 1B, *P* = 0.0232), duration (Supplementary Figure 1C, *P* = 0.0226), and principal frequency (Supplementary Figure 1D, *P* = 0.0103) of calls were reduced. In summary, VPA induced vocalization deficits at P7 and P14, which means that socially relevant behavior was damaged in the early postnatal period.

**FIGURE 2:**
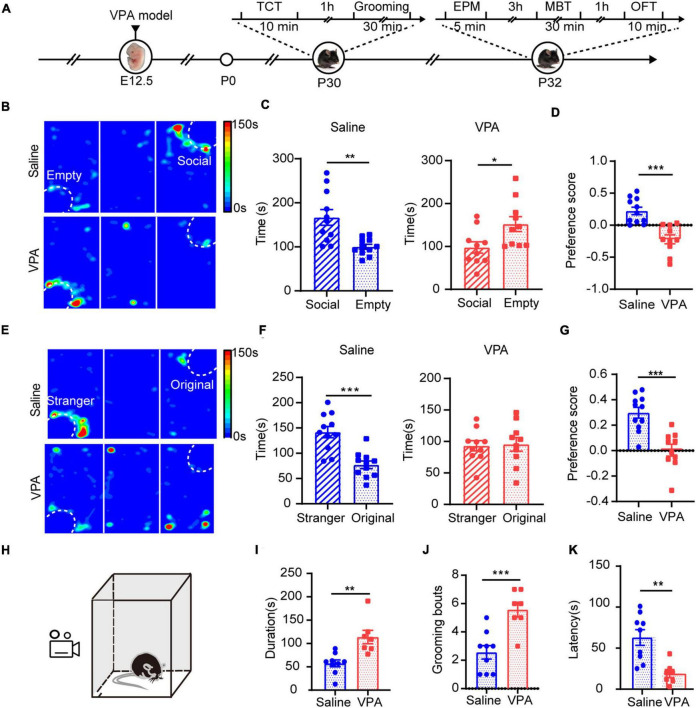
Core symptoms with autism spectrum disorders showed in VPA-exposed mice. **(A)** Timeline of behavior tests. TCT, three chamber test. EPM, elevated plus maze. MBT, marbles burying test. OFT, open field test. Sociability **(B–D)** and social preference **(E–G)** were analyzed using a three-chambered apparatus. Time spent in the social zone **(C)** and preference score **(D)** were calculated for a sociability test. Time spent in the stranger zone **(F)** and preference score **(G)** were calculated for a social preference test. Reduced social desire levels and social preference were seen in VPA group. **(H)** The schematic diagram of grooming test. Durations of grooming time **(I)**, numbers of grooming **(J)**, and latency of the first grooming **(K)** were significantly reduced in VPA-exposed mice compared to the saline-exposed mice. Data are presented as the mean ± SEM. *p < 0.5, **p < 0.01 and ***p < 0.001 vs. saline-exposed group by two-tailed unpaired Student’s *t*-test.

Next, we aimed to determine whether sociability deficits occurred in test mice exposed to VPA in their mother’s womb by using the three-chamber test to measure the sociability and social preference of the VPA mouse model. The saline-exposed mice spent more time in the social zone, indicating a stronger desire for social interaction (Figure 2C left, *n* = 11, *P* = 0.0078). In contrast, the VPA-exposed mice spent less time in the social zone (Figure 2C right, *n* = 10, *P* = 0.0121) and preferred to occupy the empty zone (Figure 2D, *P* = 0.0001), which suggests that the VPA model shows social deficits. During the social preference test, the VPA group did not indicate a preference for either social zone (Figure 2F right, *P* > 0.05). In contrast, the saline group exhibited a preference for the novel zone, socializing with the novel stimulus mouse, over the original zone, interacting with the familiar stimulus mouse (Figure 2F left, *P* = 0.0002). Moreover, to further confirm the effects of VPA on the neonatal period, we analyzed the duration of grooming behavior, the number of grooming sessions and the latency of the first grooming in the saline-exposed group and the VPA-exposed group, and the results revealed increased stereotyped behavior of VPA-exposed juvenile mice (Figures 2H–K, *n* = 9 of saline group, *n* = 7 of VPA group, *P* = 0.0022 in Figure 2I, *P* = 0.0008 in Figure 2J, *P* = 0.0021 in Figure 2K). Taken together, these results indicate that VPA exposure during the fetal period induced a typical autistic-like phenotype once mice were weaned.

Anxiety is a typical comorbidity of the autistic mouse model (Eissa et al., 2018). We tested whether anxiety was higher in our VPA-exposed mice. In the elevated plus maze (EPM) test, the VPA-exposed mice showed elevated anxiety compared with saline-exposed mice; they spent less time in the open arms and entered the open arms less (Supplementary Figures 2A,B, *n* = 9 of saline group and *n* = 9 of VPA group, *P* = 0.0011 in Supplementary Figure 2B left, *P* = 0.012 in Supplementary Figure 2B middle). In the marbles burying test (MBT), the VPA-exposed mice buried more marbles during the same time than the saline group (Supplementary Figures 2C,D, *n* = 9 of saline group and *n* = 9 of VPA group, *P* = 0.0098, *P* = 0.0177, *P* = 0.0011 at 20, 25, and 30 min). The open field test (OFT) also indicated elevated anxiety in the VPA-exposed mice (Supplementary Figures 2E,F, *n* = 9 of saline group and *n* = 9 of VPA group). The VPA-exposed mice entered the center zone less (Supplementary Figure 2F left, *P* = 0.0011) and spent less time in the center zone than the saline-exposed group (Supplementary Figure 2F middle, *P* = 0.0019), which is an anxiety-like behavior. Taken together, these results show that VPA-exposed mice exhibited increased anxiety levels and that this change occurred at least before P32.

### The Valproic Acid Mouse Model Showed Disturbances in Excitatory and Inhibitory Synapses and an Excitatory/Inhibitory Imbalance

**FIGURE 3:**
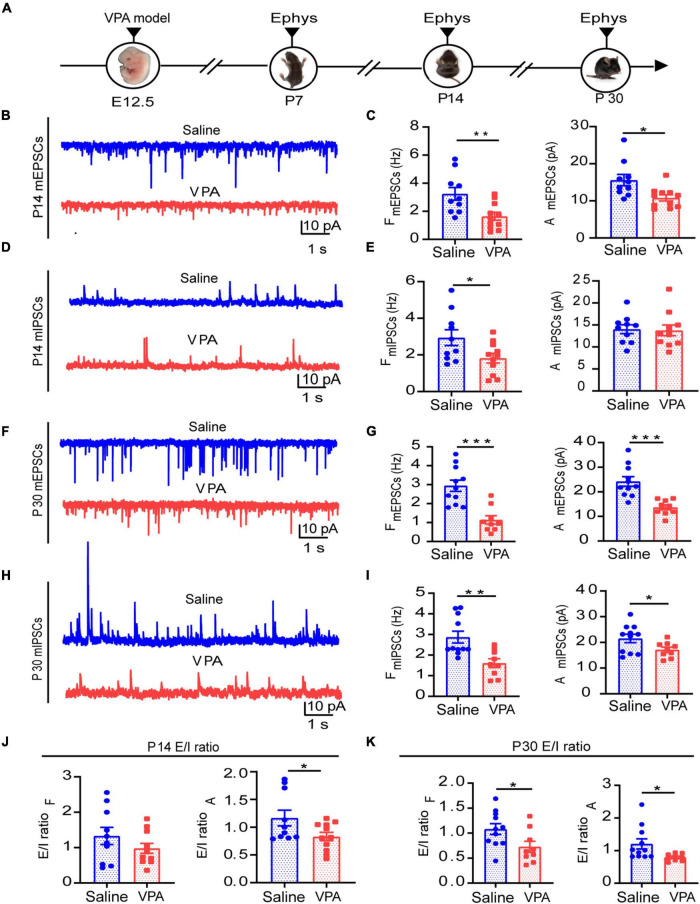
Decreased synaptic transmission and disrupted E/I balance in ACC neurons during the different development stages. **(A)**Timeline of electrophysiology recording *in vitro*. **(B)** Representative mEPSC traces in ACC pyramidal neurons at P14. **(C)** Summary data for mEPSCs frequency and peak amplitude in ACC pyramidal neurons obtained from saline-exposed group and VPA-exposed group (*n* = 10 neurons, 3 mice of saline-exposed group; *n* = 11 neurons, 3 mice of VPA-exposed group). **(D)** Representative mIPSC traces in ACC pyramidal neurons at P14. **(E)** Summary data for mIPSCs frequency and peak amplitude in ACC pyramidal neurons obtained from saline-exposed group and VPA-exposed group (*n* = 10 neurons, 3 mice of saline-exposed group; *n* = 11 neurons, 3 mice of VPA-exposed group). **(F)** Representative mEPSC traces in ACC pyramidal neurons at P30. **(G)** Summary data for mEPSCs frequency and peak amplitude in ACC pyramidal neurons obtained from saline-exposed group and VPA-exposed group (*n* = 11 neurons, 3 mice of saline-exposed group; *n* = 9 neurons, 3 mice of VPA-exposed group). **(H)** Representative mIPSC traces in ACC pyramidal neurons at P30. **(I)** Summary data for mIPSCs frequency and peak amplitude in ACC pyramidal neurons obtained from saline-exposed group and VPA-exposed group (*n* = 11 neurons, 3 mice of saline-exposed group; *n* = 9 neurons, 3 mice of VPA-exposed group). **(J)** E/I ratio of frequency and amplitude between saline-exposed group and VPA-exposed group at P14. **(K)** E/I ratio of frequency and amplitude between saline-exposed group and VPA-exposed group at P30. Data are presented as the mean ± SEM. *p < 0.05, **p < 0.01, and ***p < 0.001 vs. saline-exposed group by two-tailed unpaired Student’s *t*-test.

The E/I balance is important to maintaining the normal function of the cortex. We observed excitatory and inhibitory synaptic function via the whole-cell patch clamp technique at the same time as we observed VPA-induced abnormalities in behavior. First, we examined excitatory function at P7, since inhibitory synapses derived from local interneurons are reportedly absent or present in very small numbers in the rodent cortex until the second postnatal week (De Felipe et al., 1997). We found that excitatory synaptic transmission had already been impaired (Supplementary Figures 3A,B, *n* = 10 neurons, 3 mice of saline group; *n* = 9 neurons, 3 mice of VPA group; *P* = 0.0110). In addition, both excitatory presynaptic and postsynaptic function were impaired at P14 (Figures 3B,C, *n* = 10 neurons, 3 mice of saline group; *n* = 11 neurons, 3 mice of VPA group; *P* = 0.0065, *P* = 0.0105). And there were changes of inhibitory synaptic function and ratio of E/I balance but not significant ones (Figures 3E,J, *P* = 0.0352, *P* = 0.0457). After postnatal week 4, we found that both excitatory (Figures 3F,G, *n* = 11 neurons, 3 mice of saline group; *n* = 9 neurons, 3 mice of VPA group; *P* = 0.0002, *P* = 0.0003) and inhibitory synaptic function (Figures 3H,I, *P* = 0.0033, *P* = 0.0451) were concurrently impaired and that the E/I balance was disrupted (Figure 3K, *P* = 0.0353, *P* = 0.0318). These results further support the notion that VPA exposure during pregnancy influences synaptic function, resulting in E/I imbalance, at least in the ACC during development.

**FIGURE 4:**
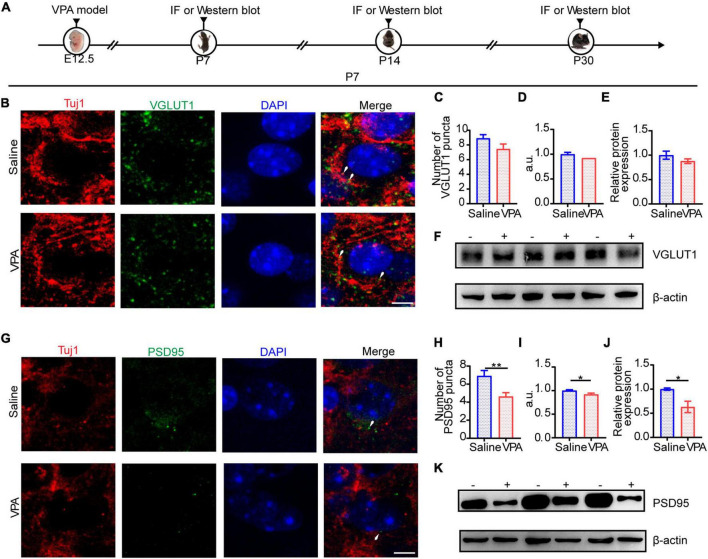
The structure of excitatory synapse in ACC was impaired at P7. **(A)** Timeline of IF (immunofluorescence) and Western blot. **(B)** IF images detecting VGLUT1 expression between saline-exposed and VPA-exposed groups. **(C,D)** IF quantitative analysis revealed expression of VGLUT1 did not change significantly between the saline-exposed group and VPA-exposed group. **(E)** Quantitative analysis of protein level of VGLUT1 of Western blot. **(F)** Representative Western blot image of VGLUT1 expression (–, saline-exposed group; +, VPA-exposed group). **(G)** IF images detecting PSD95 expression between saline-exposed and VPA-exposed groups. **(H,I)** IF quantitative analysis revealed expression of PSD95 decreased significantly of VPA-exposed group compared with the saline group. **(J)** Quantitative analysis of protein level of PSD95 of Western blot. **(K)** Representative Western blot image of PSD95 expression (–, saline-exposed group; +, VPA-exposed group). Scale bar = 5 μm. Data are presented as the mean ± SEM. *p < 0.05 and **p < 0.01 vs. saline-exposed group by two-tailed unpaired Student’s *t*-test.

Given that profound synaptic dysfunction is the major cause of E/I imbalance in the ACC, we sought to test whether the protein levels of typical synaptic components were changed. Glutamate and GABA mediate most of the excitatory and inhibitory synaptic transmission in the mammalian brain, respectively; VGLUT1, which represents excitatory presynaptic transmission, is expressed mainly in the telencephalon (Fattorini et al., 2009), as well as PSD95, a major synaptic protein that clusters glutamate receptors and is critical for synaptic plasticity (Bustos et al., 2017). At P7, we tested the expression of VGLUT1 (Figures 4C,D, cells = 11 of 2 mice in each group; Figures 4E,F *n* = 3 mice in each group) and PSD95 (Figures 4H,I, cells = 11 of 2 mice in each group, *P* = 0.0058, *P* = 0.0343; Figures 4J,K, *n* = 3 mice in each group, *P* = 0.0365) by immunocytochemistry and Western blotting. The results showed that the expression of PSD95 was significantly decreased compared with that of VGLUT1.

**FIGURE 5:**
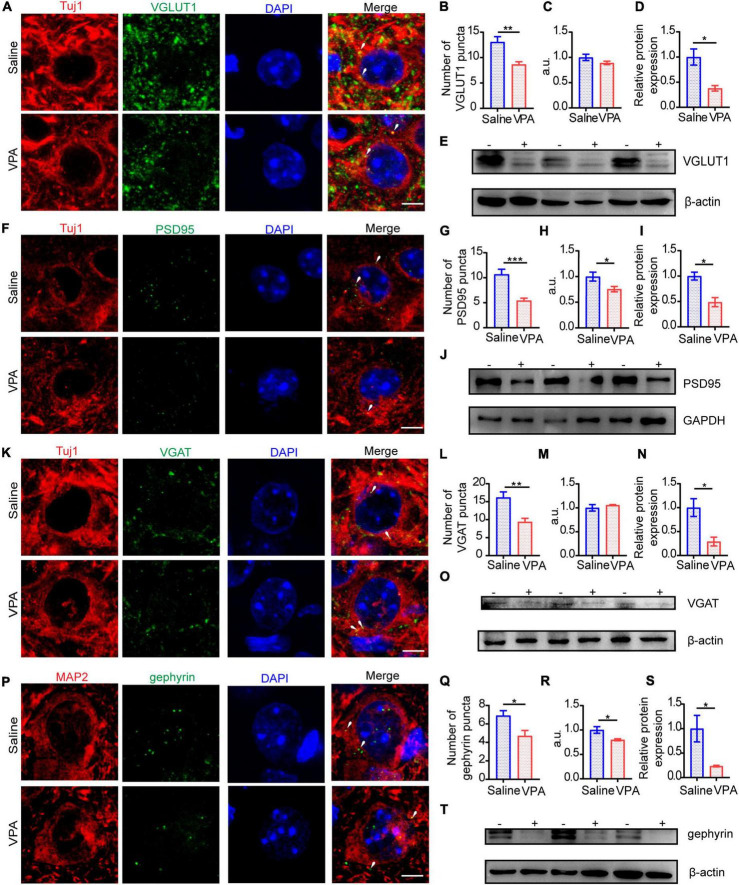
The structure of excitatory synapses and inhibitory synapses in ACC were impaired at P14. **(A)** IF images detecting VGLUT1 expression between saline-exposed and VPA-exposed groups. **(B,C)** IF quantitative analysis revealed expression of VGLUT1 changed significantly between saline-exposed group and VPA-exposed group. **(D)** Quantitative analysis of protein level of VGLUT1 of Western blot. **(E)** Representative Western blot image of VGLUT1 expression (–, saline-exposed group; +, VPA-exposed group). **(F)** IF images detecting PSD95 expression between saline-exposed and VPA-exposed groups. **(G,H)** IF quantitative analysis revealed expression of PSD95 changed significantly between saline-exposed group and VPA-exposed group. **(I)** Quantitative analysis of protein level of PSD95 of Western blot. **(J)** Representative Western blot image of PSD95 expression (–, saline-exposed group; +, VPA-exposed group). **(K)** IF images detecting VGAT expression between saline-exposed and VPA-exposed groups. **(L,M)** IF quantitative analysis revealed expression of VGAT changed between saline-exposed group and VPA-exposed group. **(N)** Quantitative analysis of protein level of VGAT of Western blot. **(O)** Representative Western blot image of VGAT expression (–, saline-exposed group; +, VPA-exposed group). **(P)** IF images detecting gephyrin expression between saline-exposed and VPA-exposed groups. **(Q,R)** IF quantitative analysis revealed expression of gephyrin changed between saline-exposed group and VPA-exposed group. **(S)** Quantitative analysis of protein level of gephyrin of Western blot. **(T)** Representative Western blot image of gephyrin expression (–, saline-exposed group; +, VPA-exposed group). Scale bar = 5 μm. Data are presented as the mean ± SEM. *p < 0.05, **p < 0.01, and ***p < 0.001 vs. saline-exposed group by two-tailed unpaired Student’s *t*-test.

**FIGURE 6:**
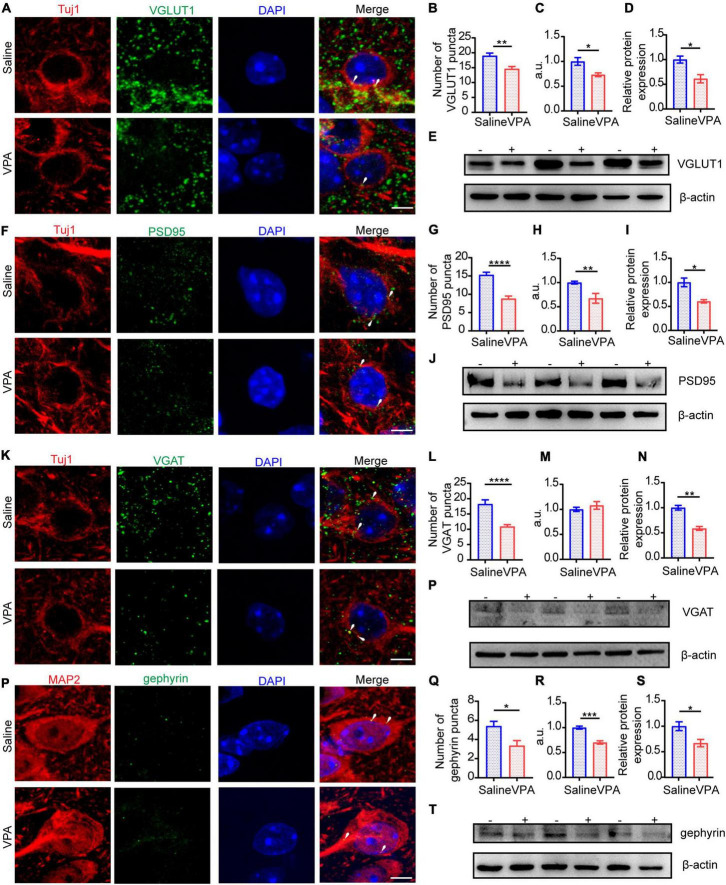
The structure of excitatory synapses and inhibitory synapses in ACC were impaired significantly at the late period of development. **(A)** IF images detecting VGLUT1 expression between saline-exposed and VPA-exposed groups. **(B,C)** IF quantitative analysis revealed expression of VGLUT1 changed significantly between saline-exposed group and VPA-exposed group. **(D)** Quantitative analysis of protein level of VGLUT1 of Western blot. **(E)** Representative Western blot image of VGLUT1 expression (–, saline-exposed group; +, VPA-exposed group). **(F)** IF images detecting PSD95 expression between saline-exposed and VPA-exposed groups. **(G,H)** IF quantitative analysis revealed expression of PSD95 changed significantly between saline-exposed group and VPA-exposed group. **(I)** Quantitative analysis of protein level of PSD95 of Western blot. **(J)** Representative Western blot image of PSD95 expression (–, saline-exposed group; +, VPA-exposed group). **(K)** IF images detecting VGAT expression between saline-exposed and VPA-exposed groups. **(L,M)** IF quantitative analysis revealed expression of VGAT changed between saline-exposed group and VPA-exposed group. **(N)** Quantitative analysis of protein level of VGAT of Western blot. **(O)** Representative Western blot image of VGAT expression (–, saline-exposed group; +, VPA-exposed group). **(P)** IF images detecting gephyrin expression between saline-exposed and VPA-exposed groups. **(Q,R)** IF quantitative analysis revealed expression of gephyrin changed between saline-exposed group and VPA-exposed group. **(S)** Quantitative analysis of protein level of gephyrin of Western blot. **(T)** Representative Western blot image of gephyrin expression (–, saline-exposed group; +, VPA-exposed group). Scale bar = 5 μm. Data are presented as the mean ± SEM. *p < 0.05, **p < 0.01, ***p < 0.001, and ****p < 0.0001 vs. saline-exposed group by two-tailed unpaired Student’s *t*-test.

At P14 and P30, the protein expression of excitatory and inhibitory synapses was severely decreased (Figures 5, 6). The expression of VGLUT1 was also decreased on these 2 days (Figures 5B,C, cells = 13 of 2 mice in saline group and cells = 12 of 2 mice in VPA group, *P* = 0.0013; Figures 5D,E, *n* = 3 mice in each group, *P* = 0.0216; Figures 6B,C, cells = 11 of 2 mice in each group, *P* = 0.0012, *P* = 0.0204; Figures 6D,E *n* = 3 mice in each group, *P* = 0.0236). At P30, the expression of PSD95 was markedly reduced (Figures 6G,H, cells = 10 of 2 mice in saline group and cells = 11 of 2 mice in VPA group, *P* < 0.0001, *P* = 0.0078; Figures 6I,J, *n* = 3 mice in each group, *P* = 0.0151) compared with that at P14 (Figures 5G,H, cells = 10 of 2 mice in saline group and cells = 9 of 2 mice in VPA group, *P* = 0.0003, *P* = 0.0443; Figures 5I,J, *n* = 3 mice in each group, *P* = 0.0117). To evaluate the cause of the inhibitory synapse abnormalities, we observed the protein levels of VGAT and gephyrin, two markers that reflect inhibitory synaptic function. GABA is transferred into synaptic vesicles by the vesicular transporter VGAT, which is expressed throughout the brain; VGAT is localized to the presynaptic element forming inhibitory symmetric synapses (Fattorini et al., 2009). Gephyrin is a 93 kDa protein initially identified as a tubulin-binding molecule that is localized at the inhibitory postsynaptic locus; by interacting with various proteins, gephyrin further regulates synapse formation and plasticity (Pizzarelli et al., 2020). Thus, gephyrin exerts powerful control over network excitability and oscillatory behavior crucial for information processing. Abnormal expression of VGAT (Figures 5L,M, cells = 12 of 2 mice in saline group and cells = 10 of 2 mice in VPA group, *P* = 0.0022; Figures 5N,O, *n* = 3 mice in each group, *P* = 0.0274) and gephyrin (Figures 5Q,R, cells = 11 of 2 mice in saline group and cells = 10 of 2 mice in VPA group, *P* = 0.0120, *P* = 0.0471; Figures 5S,T, *n* = 3 mice in each group, *P* = 0.0480) arose at P14, and after 2 weeks, these reduction in expression became more pronounced not only of VGAT (Figures 6L,M, cells = 12 of 2 mice in saline group and cells = 13 of 2 mice in VPA group, *P* < 0.0001; *n* = 3 mice in each group, *P* = 0.0025) but also of gephyrin (Figures 6Q,R, cells = 10 of 2 mice in saline group and cells = 11 of 2 mice in VPA group, *P* = 0.0116, *P* = 0.0007; Figures 6S,T, *n* = 3 mice in each group, *P* = 0.0413). The aberrant expression levels of VGAT and gephyrin are likely to damage inhibitory synaptic function. These data indicate that the E/I imbalance caused by impeding excitatory and inhibitory synapses might involve in following aspects, such as function, structure and plasticity and the negative impacts on E/I balance induced by VPA exposure at pregnancy become more severe with neural development.

### Reduced Brain-Derived Neurotrophic Factor Expression Is a Potential Key Factor for Excitatory/Inhibitory Imbalance in the Valproic Acid Mouse Model

**FIGURE 7:**
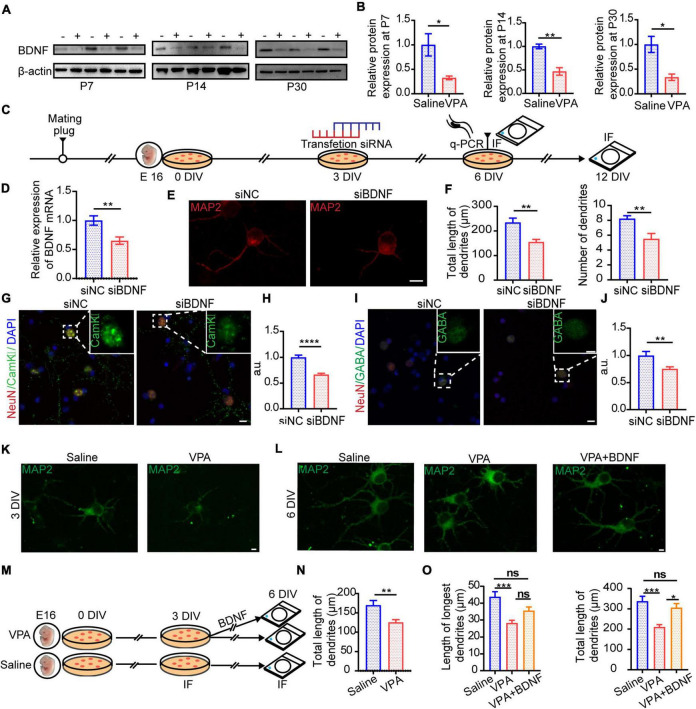
BDNF expression changed in VPA-exposed group in vivo and it influenced excitatory and inhibitory synapses developmental plasticity *in vitro*. **(A)** Representative Western blot images of BDNF expression (–, saline-exposed group; +, VPA-exposed group) at P7, P14, and P30. **(B)** Quantitative analysis of protein levels of BDNF of Western blot. *p < 0.05 and **p < 0.01 vs. saline-exposed group by two-tailed unpaired Student’s *t*-test. **(C)** Timeline of siRNA transfection to primary cell culture. **(D)** qRT-PCR result showed siRNA transfection in primary cell culture silencing BDNF mRNA expression. **(E)** Typical IF images of primary cell culture exposed to siNC (negative control, left) and siBDNF (right). Scale bar = 10 μm. **(F)** Quantification of morphological difference. Total length of dendrites (left) and numbers of dendrites (right) were decreased in siBDNF group. **(G)** Typical IF images of CaMKII expression of primary cell culture exposed to siNC (left) and siBDNF (right) after 12 DIV (Scale bar = 10 μm). Insets are the enlarged image of dashed areas in each figure (Scale bar = 5 μm). **(H)** IF quantitative analysis revealed expression of CaMKII changed significantly between siNC and siBDNF group. **(I)** Typical IF images of GABA expression of primary cell culture exposed to siNC (left) and siBDNF (right) after 12 DIV (Scale bar = 10 μm). Insets are an enlarged image of dashed areas in each figure (Scale bar = 5 μm). **(J)** IF quantitative analysis revealed expression of GABA changed significantly between siNC and siBDNF group. *p < 0.05, **p < 0.01, and ****p < 0.0001 vs. siNC group by two-tailed unpaired Student’s *t*-test. **(K)** Typical IF images of primary cell culture from embryos exposed to saline (left) or VPA (right) prenatally after 3 DIV. Scale bar = 5 μm. **(L)** Typical IF images of primary cell culture from embryos exposed to saline (left) or VPA (middle) or VPA+BDNF (BDNF added in 3 DIV, right) prenatally after 6 DIV. Scale bar = 5 μm. **(M)** Timeline of BDNF treatment to primary cell culture. **(N)** Quantification of the total length of dendrites between embryos exposed to saline or VPA prenatally after 3 DIV. **(O)** Quantification of length of longest dendrites (left) and total length of dendrites (right) between saline or VPA or VPA+BDNF groups after 6 DIV. Measurement in length and number of dendrites used Imaris 7.4.2. **(N)** **p < 0.01 vs. saline-exposed group by two-tailed unpaired Student’s *t*-test except. **(O)** *p < 0.05 and ***p < 0.001 vs. saline-exposed group by one-way ANOVA. Data are presented as the mean ± SEM.

Our present results revealed that not only excitatory synapses but also inhibitory synapses exhibited impairment from VPA exposure (Figures 4–6). We hypothesized that there might be a critical factor that concurrently influences glutamatergic pyramidal neurons and GABAergic interneurons, leading to the E/I imbalance. Brain-derived neurotrophic factor (BDNF), a member of the neurotrophin family of neurotrophic factors, is a protein that regulates the neural differentiation, survival, and development. In addition, previous studies have indicated that this growth factor is a key element, especially for the early induction of autism (Meng et al., 2017; Win-Shwe et al., 2018), as well as in other studies conducted on Angelman and Rett syndromes, both of which belong to the spectrum of neurological disorders associated with autism (Li and Pozzo-Miller, 2014). It seems that synaptic growth factors play a key role in the development and plasticity of neuronal circuits. Taken together, the above results indicate that BDNF might be the key factor in the progression of autism spectrum disorder. In our experiment, BDNF expression decreased notably not only in the early stage but also in later stages of development (Figures 7A,B, *n* = 3 mice in each group, *P* = 0.0429, *P* = 0.0046, *P* = 0.0195), confirming our hypothesis. Because BDNF was downregulated in the ACC of the VPA-exposed group and exogenous BDNF facilitated natural neuron development, we applied siRNA to knock down BDNF in primary culture and the diagram showed experimental procedure (Figure 7C). Knockdown of BDNF at the mRNA (Figure 7D, P = 0.0068) level also reduced dendrite development (Figures 7E,F, cells = 9 in siNC group and cells = 8 in siBDNF group, *P* = 0.0020, *P* = 0.0042) and limited growth of glutamatergic pyramidal neurons (Figures 7G,H, cells = 16 of siNC group and cells = 14 of siBDNF group, *P* < 0.0001) and GABAergic interneurons (Figures 7I,J, cells = 12 of siNC group and cells = 13 of siBDNF group, *P* = 0.0060). To further characterize the role of BDNF in dendrite development, we examined the effects of prenatal exposure to VPA on dendritic growth and development, as indices of neuronal maturation, in primary cortical neurons (Figures 7K–O). Exposure to VPA at E12.5 caused significant decreases in the total length of dendrites after 3 days *in vitro* (DIV) (Figures 7K,N, cells = 10 of saline group and cells = 9 of VPA group, *P* = 0.0066). To investigate the role of BDNF in regulating the development of dissociated cortical neurons, cultures were treated with 500 ng/mL BDNF and then compared with untreated controls. After 6 DIV, primary cells exposed to VPA during the prenatal period exhibited abnormalities in dendritic development, but endogenous BDNF rescued this developmental dysfunction (Figures 7L,O, O left, cells = 9 in each group *P* = 0.0004; O right, cells = 8 in each group *P* = 0.0007 *P* = 0.0116). Taken together, our results suggest that decreased expression of BDNF, as a result of VPA exposure during gestation, might influence synaptic development and plasticity to disrupt the E/I balance.

### Social Behavior and Synaptic Function Were Rescued by Brain-Derived Neurotrophic Factor Intervention in the Valproic Acid Mouse Model

**FIGURE 8:**
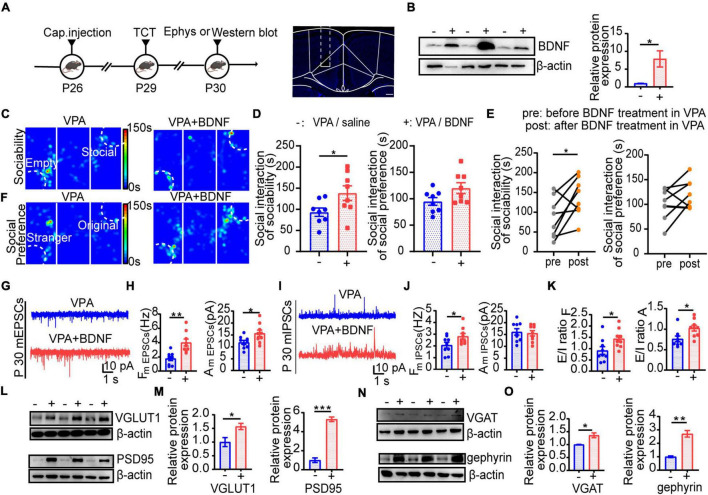
BDNF improved E/I balance to remit social deficit by regulating synaptic function. **(A)** Left: scheme of BDNF treatment and tests applied after treatment. Right: scheme of ACC area target with BDNF injection. Scale bar = 200 μm. **(B)** Left: representative Western blot image of BDNF expression (–, VPA/saline group; +, VPA/BDNF group). Right: quantitative analysis of protein level of BDNF of Western blot. **(C)** Representative heat maps showing alteration in locomotion and sociability in three chamber. Left: VPA/saline group; right: VPA/BDNF group. **(D)** Left: time spent in the social zone between VPA/saline group and VPA/BDNF group. Right: time spent in the stranger zone between VPA/saline group and VPA/BDNF group. **(E)** Left: time spent in social zone of VPA-exposed group before and after BDNF treatment. Right: time spent in stranger zone of VPA-exposed group before and after BDNF treatment. **(F)** Representative heat maps showing alteration in locomotion and social preference in three chamber. Left: VPA/saline group; right: VPA/BDNF group. **(G)** Representative mEPSC traces in ACC pyramidal neurons at P30 after treatment (VPA: VPA/saline group; VPA+BDNF: VPA/BDNF group). **(H)** Summary data for mEPSCs frequency and peak amplitude in ACC pyramidal neurons after treatment (–, VPA/saline group; +, VPA/BDNF group) (*n* = 9 neurons, 3 mice in each groups). **(I)** Representative mIPSC traces in ACC pyramidal neurons at P30 after treatment (VPA: VPA/saline group; VPA+BDNF: VPA/BDNF group). **(J)** Summary data for mIPSCs frequency and peak amplitude in ACC pyramidal neurons after treatment (–, VPA/saline group; +, VPA/BDNF group) (*n* = 9 neurons, 3 mice in each groups). **(K)** Showed E/I ratio of frequency and amplitude after treatment (–, VPA/saline group; +, VPA/BDNF group). **(L)** Representative Western blot images of VGLUT1 and PSD95 expression (–, VPA/saline group; +, VPA/BDNF group). **(M)** Left: quantitative analysis of protein level of VGLUT1 of Western blot. Right: quantitative analysis of protein level of PSD95 of Western blot. **(N)** Representative Western blot images of VGAT and gephyrin expression (–, VPA/saline group; +, VPA/BDNF group). **(O)** Left: quantitative analysis of protein level of VGAT of Western blot. Right: quantitative analysis of protein level of gephyrin of Western blot. Data are presented as the mean ± SEM. *p < 0.05, **p < 0.01, and ***p < 0.001 between VPA/saline group and VPA/BDNF group by two-tailed unpaired Student’s *t*-test except **(E)** was conducted with two-tailed paired Student’s *t*-test.

Theorizing that the profound deficit in BDNF expression in the ACC might be a pivotal factor influencing synapse development, we injected BDNF by implanting minipumps that administered 114 μL BDNF (0.0013 μg/μL) for three consecutive days (starting at P26) in the ACC, followed by behavioral tests and synaptic activity measurements by the patch clamp technique at P29 and P30 (Figure 8A). The BDNF expression increased after this injection (Figure 8B, *n* = 3 mice in each group, *P* = 0.0387). Interestingly, time spent in the social chamber was increased, which means that the social deficits were decreased (Figures 8C–E, *n* = 8 mice in each group, *P* = 0.0419 of Figure 8D left; *n* = 8 mice in each group, *P* = 0.0364 of Figure 8E left), but there was no obvious increase in social selectivity (Figures 8D–F, *n* = 8 mice in each group, *P* > 0.05 of Figure 8D right; *n* = 8 mice in each group, *P* > 0.05 of Figure 8E right). Moreover, the function of excitatory synapse was obviously altered (Figures 8G,H, *n* = 9 in each group, *P* = 0.0011, *P* = 0.0274) compared with that of the inhibitory synapses (Figures 8I,J, *n* = 9 in each group, *P* = 0.0434, *P* > 0.05), and the E/I balance was rescued while this pharmacological enhancement was sustained (Figure 8K, *n* = 9 in each group, *P* = 0.0297, *P* = 0.0155). To further confirm that these changes were involved in the changes in synaptic function, we observed synaptic marker protein expression (Figures 8L–O). Although all these synaptic marker proteins had higher expression in the saline group than the VPA group, the postsynaptic proteins PSD95 and gephyrin had even greater increases (Figures 8L–O, *n* = 3 mice in each group, *P* = 0.0455, *P* = 0.0002 of Figure 8M; *P* = 0.0189, *P* = 0.0025 of Figure 8O). These results corroborate the notion that normal synaptic function is necessary to maintain the E/I balance to properly regulate the ACC, and the effect of exogenous BDNF may provide a new pharmacological intervention target for reducing disturbances in behavior in ASD.

## Discussion

Valproic acid administration to pregnant rats and mice during gestation leads to autistic-like symptoms in the offspring, and this method is widely used as an animal model for autism (Morales-Navas et al., 2020; Tsuji et al., 2020). USVs can be observed during the neonatal period, as these isolation-induced calls serve to elicit retrieval behavior from lactating mice (Gzielo et al., 2020); thus, they may serve as communication signals between the mother and pups. During the early postnatal period, changes in USVs typically occur in this autism animal model. Previous studies have mainly focused on rats and ICR mice, but research on C57BL/6 mice is lacking. USVs change between P7 and P11, and a study showed that the peak of several calls appeared between P6 and P8 (Wang et al., 2020). Therefore, we explored the VPA-exposed autism model in C57BL/6 mice and found a significant reduction in USVs at P7 (Figure 1) that persisted to P14 (Supplementary Figure 1), which indicates that socially relevant behavior abnormalities occurred early in development. Other strains of mice, such as ICR mice or Swiss albino mice, have been reported to exhibit social behavior deficits, increased grooming, and anxiety behavior approximately 3–4 weeks after birth (Al-Amin et al., 2015; Gao et al., 2019). Besides that, VPA also was found to induce ASD like phenotype in the marmoset (Watanabe et al., 2021). In the present study, mice exhibited a similar pattern, indicating the feasibility of different subgroups of mice for this autism mouse model and confirming the success of our model, which suggested that exposure to VPA during pregnancy impacts offspring from the early postnatal stage to adolescence. According to our project design, we observed social behavior at P30 and anxiety status at P32. But these changes might occur in an earlier development stage and it may continue into the adult stage (Al-Amin et al., 2015; Gao et al., 2019). It is better to assess social related behavior between P14 and P30. However, USVs reduced lots after P14. And the mice around P21 are very active, which is not very suitable for this test. Some new behavioral paradigm may be needed for evaluating the social function of the mice around P21–P28.

Cortical circuits in the brain are refined by experience during critical periods early in postnatal life and these critical periods are regulated by the balance of excitatory and inhibitory (E/I) neurotransmission in the brain during development (LeBlanc and Fagiolini, 2011). Numerous studies have investigated the influence of VPA exposure during pregnancy on excitatory synaptic development (Kim et al., 2013; Fueta et al., 2018) and GABAergic synaptic signalings (Hou et al., 2018). However, fewer have focused on holistic changes in the E/I balance. Some studies with mutant mouse models have demonstrated an E/I imbalance (Mao et al., 2015; Antoine et al., 2019). Environmental factors, such as VPA exposure, are especially important and can lead to autism. In addition, expression of glutamatergic or GABAergic proteins was observed in VPA-exposed autism models (Banerjee et al., 2013; Chau et al., 2017; Lenart et al., 2020). However, both excitatory and inhibitory synaptic functions are needed to regulate the E/I balance, and this balance determines the condition of local cortical circuits. Therefore, in our research, we explored excitatory and inhibitory synaptic function concurrently in a VPA-exposed mouse model, emphasizing alterations in E/I balance, and the results showed that social deficits were observed when E/I imbalance occurred. At P14, we observed that both excitatory synapses and inhibitory synapses reduced transmission (Figure 3), and this reduction suggests that GABAergic interneurons exhibited dysplasia during early development. However, the E/I balance was not significantly altered (Figure 3J). After weaning, VPA-exposed mice exhibited representative abnormal autism-like social behavior (Figure 2) and a concurrent prominent E/I imbalance (Figure 3K). Further research showed that excitatory and inhibitory synaptic structure was one of the causes of this imbalance (Figures 5, 6).

The alteration in the balance between inhibitory and excitatory synaptic transmission is emerging as a fundamental principle underlying a variety of neuropsychiatric and neurodevelopmental disorders, such as ASD and epilepsy. Using the ratio of mEPSCs over mIPSCs to describe the change of E/I balance is a common method (Bassetti et al., 2021; Huang et al., 2021). It can show the whole view of the excitatory synaptic inputs and inhibitory synaptic inputs from the postsynaptic neuron. And it also is straightforward from the technological perspective. However, mini PSCs did have some limitations. For example, it can only reflect the basal synaptic transmission. But activities dependent synaptic transmission is more common during the neuron executing the function. Evoked PSCs are frequently used for measuring the activities related to synaptic function. However, the response of the evoked PSCs highly relies on the specificity of presynaptic inputs, which depend on the position of the stimulating electrode. For example, in ACC, there are many types of GABAergic interneurons, just like parvalbumin^+^ (PV) interneurons, somatostatin^+^ (SST) interneurons and vasoactive intestinal peptide^+^ (VIP) interneurons and they have special electrophysiological properties, anatomical properties and connectivity which could increase the heterogeneity of evoked IPSCs. Our present work suggested that E/I imbalance happened in the VPA mice. We still need to tell part what kind of synaptic input produced this deficit. We would like to explore it with evoked PSCs with a more specific stimulating method, such as optogenetics.

For the mEPSCs in cortex of VPA-exposed autism model, one report showed that general hyper-connectivity after P10 in the auditory cortex and mEPSC amplitude of subplate neurons was dramatically increased (Nagode et al., 2017). It is similar with previous clinical research showing that sensory abnormalities are a very frequent feature in some children with ASD (Posar and Visconti, 2018). Three main sensory patterns have been described in autism spectrum disorder: hypo-responsiveness, hyper-responsiveness, and sensory seeking. Auditory is one of sensory capacity. The abnormal connection might cause the abnormity of auditory in autism. It has been reported that dysfunction of the somatosensory cortex leads to sensory hyper-reactivity in a *Shank3* mouse model of ASD (Chen et al., 2020), so it is quite possible that auditory, acting as one of the sensory abilities, become more sensitive in autism patients. However, we have different observations in the ACC area. Our previous results suggested that ACC is a cortical region participating in regulating the social behavior of *Shank3* ASD mouse model (Guo et al., 2019). And mEPSCs amplitude decreased in this *Shank3* mouse model of ACC which is consistent with our results of ACC in VPA-exposed mouse model. Our results and Nagode’s suggested that different cortical regions play a different role in controlling behavior, cognitive competence, and emotion. Based on the above analysis, the VPA-exposed induced autism mouse model reflected neural development disease traits. It has been proposed that alteration of the expression and/or timing of critical period circuit refinement in primary sensory brain areas may significantly contribute to autistic phenotypes, including cognitive and behavioral impairments (LeBlanc and Fagiolini, 2011), which indicates the importance to explore the time course of disease occurrence and development. We found distinct E/I imbalances at different time points, which could provide a reference for selecting the appropriate time window for clinical interventions.

According to morphological structure change in the VPA-expose model, one proposed that VPA-induced marmoset model of ASD shows increased synaptogenesis at postnatal 6 months old (Watanabe et al., 2021). Although we have not observed spine density in our research, we think the spine density might decrease with the development stage based on our previous results. There are several differences between Watanabe and our work. First, they used the non-human primates as the object of study which is different from our research object; and age is another factor that we need to think about; the third difference is the brain region of research between our work and the work of the marmoset model. We focused on the anterior cingulate cortex which belongs to Brodmann area 24, 25, 32, 33 (Palomero-Gallagher et al., 2009, 2019) but the work of the marmoset model observed layer 3 pyramidal neurons of Brodmann area 8b/9. These differences may have influenced the detailed results. In our research, we pay attention to the change of both excitatory synapse and inhibitory synapse simultaneously. And usually, inhibitory synapses did not form the spine with postsynaptic neurons. So it is hard to measure the excitatory synapses and inhibitory synapses structure at the spine level simultaneously. Based on literature reports and the electrophysiological and biochemical results in our results, we speculate spine density in ACC may decrease more seriously with development probably. Furthermore, according to some reports, the cortical spine density of mice in VPA-exposed model showed decreased or no difference in the adult stage (Hara et al., 2017; Mahmood et al., 2018). It is worthy to study in our future research.

Brain-derived neurotrophic factor, a member of the neurotrophin family of growth factors, modulates nerve growth during neuronal development and plasticity (Nawa et al., 1994). The chronic presence of BDNF enhances the formation and functional maturation of glutamatergic and GABAergic synapses (Mizuno et al., 1994). The presence of BDNF is required for proper cycling of synaptic vesicles during high-frequency stimulation and is one of the crucial mediators of long-term potentiation (LTP) at glutamatergic and GABAergic synapses in the central nervous system (CNS) (Gottmann et al., 2009). BDNF may play a key role in the concurrent enhancement in the function and structure of excitatory and inhibitory synapses, improving the E/I balance. Moreover, BDNF expression levels in the ACC were decreased in our study (Figures 7A,B), which is consistent with other studies that have reported that BDNF expression decreases in the prefrontal cortex, hippocampus, and other regions of the brain in a VPA-exposed autism model (Chau et al., 2017; Alo et al., 2021). In addition, we also observed that steady administration of BDNF into the ACC during the severe period of E/I imbalance improved social defects (Figures 8C–E). The probable mechanism of BDNF is through potentiating synaptic transmission and regulating synaptic function, similar to other members of the neurotrophin family of growth factors. Other mechanisms include binding of BDNF to the TrkB receptor, which increases the accumulation of synaptic vesicles at the active zone in the presynaptic region, local translation at the synapse and upregulation of gene expression, and changes in the synaptic proteome and cytoskeleton (Leal et al., 2014). In addition, BDNF regulates mRNA transportation in dendrites and changes the neuronal proteome. The probable mechanism by which BDNF adjusts GABAergic interneuron and inhibitory synaptic function is by upregulating GAD65/67 mRNA levels according to increased synaptogenesis and expression of the K^+^/Cl^–^ cotransporter KCC2 (Aguado et al., 2003) and by increasing the number of GABAAR clusters and the percentage of synaptically localized GABAAR clusters (Elmariah et al., 2004). Moreover, the application of BDNF to immature neuronal cultures from the rat hippocampus increased the protein levels and clustering of gephyrin (Gonzalez, 2014). In this VPA-induced autism model, the complex mechanism by which BDNF regulates excitatory synapses and inhibitory synapses should be explored in further studies. Based on our search and other reports (Bryn et al., 2015; Armeanu et al., 2017), a growing number of studies have focused on the link between BDNF and autism. Autism could be a disease where there is a role for several neurotransmitters including BDNF. So, it would be appropriate to measure brain acetylcholine, GABA, dopamine, serotonin levels and correlate the same to the levels of BDNF, and decipher the relationship between various neurotransmitters and the development of autism in our later studies. The study of neurotransmitters may provide a potential for translation into new treatments for the disorder (Rojas, 2014). In addition, according to previous studies, the occurrence and development of autism are also related to inflammatory factors (Vargas et al., 2005), so this could also serve as a new direction for our study of mouse models of autism.

## Conclusion

In summary, we showed that when exposed to VPA treatment during pregnancy, pups exhibited autism-like behavior after birth, which might be related to the E/I imbalance in the ACC resulting from aberrant excitatory and inhibitory synaptic development and plasticity, and the progression of this dysfunction increases with postnatal neural development. We propose BDNF intervention in a crucial time window may facilitate excitatory and inhibitory synaptic development concurrently to improve the E/I balance and rescue behavioral deficits.

## Data Availability Statement

The raw data supporting the conclusions of this article will be made available by the authors, without undue reservation.

## Ethics Statement

The animal study was reviewed and approved by the Institutional Animal Care and Use Committee (IACUC) of the Fourth Military Medical University.

## Author Contributions

WW and SW designed the experimental plans. CQ, HM, and GM performed the behavioral part of the study. CQ, AC, EH, and QX did the molecular biological experiment. CQ and KR did the electrophysiology recording. CQ and JG analyzed the data. WW, SW, and CQ wrote and revised the manuscript. All authors contributed to the article and approved the submitted version.

## Conflict of Interest

The authors declare that the research was conducted in the absence of any commercial or financial relationships that could be construed as a potential conflict of interest.

## Publisher’s Note

All claims expressed in this article are solely those of the authors and do not necessarily represent those of their affiliated organizations, or those of the publisher, the editors and the reviewers. Any product that may be evaluated in this article, or claim that may be made by its manufacturer, is not guaranteed or endorsed by the publisher.

## Footnotes

1
https://github.com/DrCoffey/DeepSqueak
