# Supplementary material for: Excitatory and Inhibitory Synaptic Imbalance Caused by Brain-Derived Neurotrophic Factor Deficits During Development in a Valproic Acid Mouse Model of Autism
Source: Front Mol Neurosci. 2022 Apr 6;15:860275. doi: 10.3389/fnmol.2022.860275 (PMC9019547; doi:10.3389/fnmol.2022.860275)
Supplement: Supplementary file 1 [file Data_Sheet_1.docx]

Supplementary Material

# Supplementary Figures


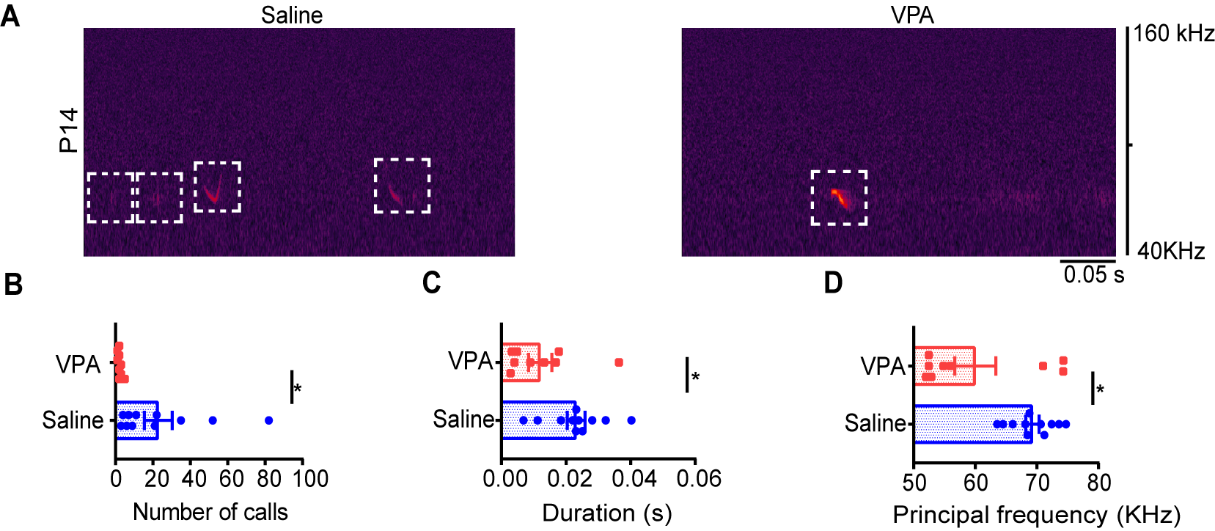


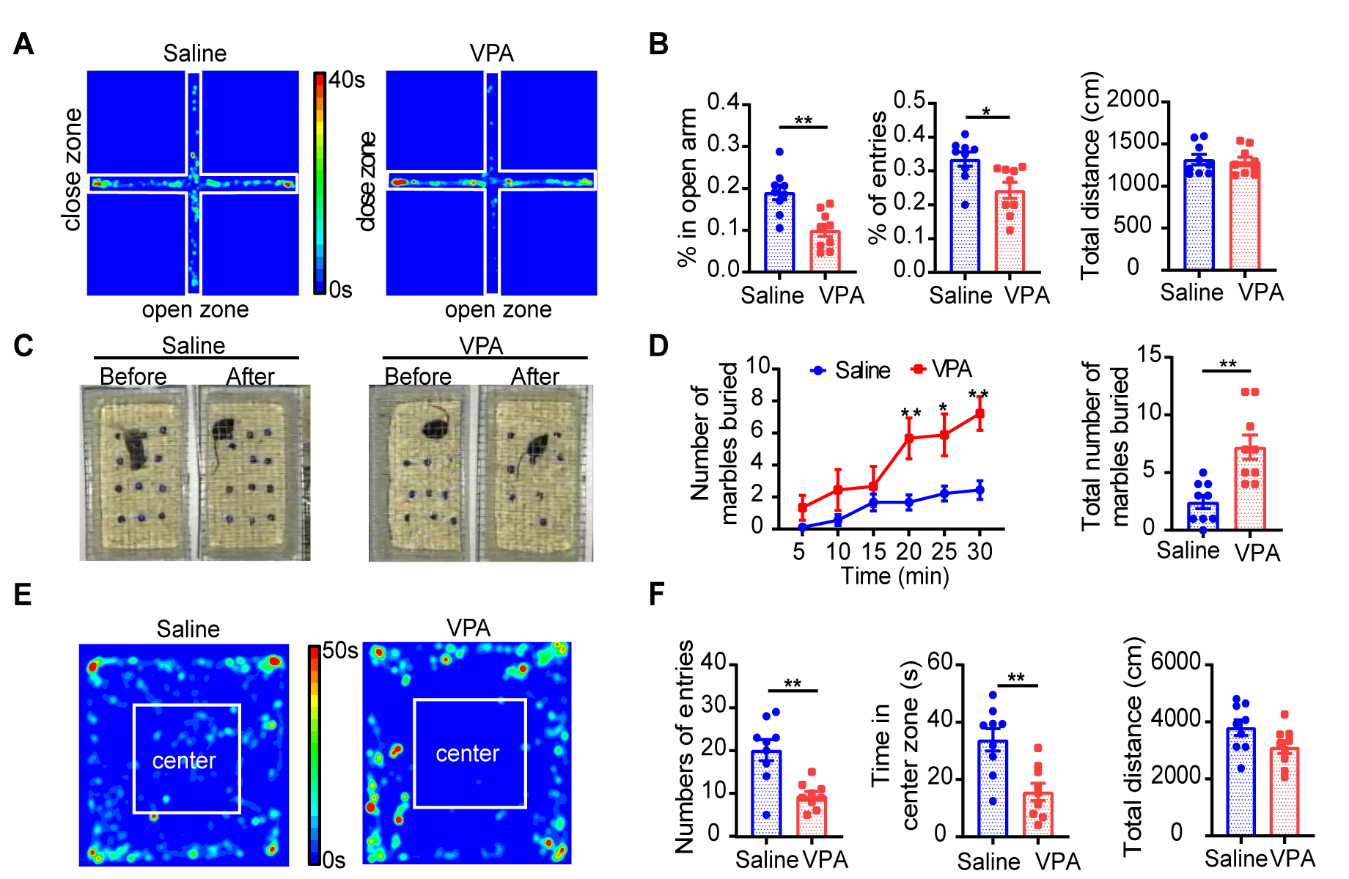
**Figure S1.** VPA-exposed pups expressed decreasing calls of attention at P14. (A) Left: canonical waveforms of USVs from the saline-exposed group. Right: canonical waveforms of USVs from the VPA-exposed group. Data are presented (B) number of calls, (C) duration, (D) principal frequency of the calls (Saline: n=11; VPA: n=9). Data are presented as the mean ± s.e.m. **p*<0.05 vs. saline-exposed group by two-tailed unpaired Student’s t-test.


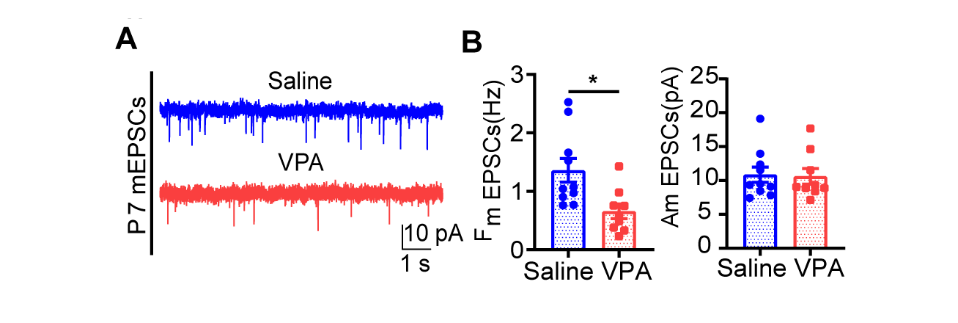
**Figure S2.** Elevated anxiety in VPA-exposed mice. (A) Representative heat map showing alteration in locomotion and exploratory behavior in EPM (distance traveled in open zone vs closed zone). (B) Left: percentage of time spent in open arm; Middle: percentage of number of entries into open arm; Right: total distance. (C) The representative screenshots of marbles burying test between saline-exposed group and VPA-exposed group. (D) Left: time course showed the numbers of marbles buried per 5 min. Right: total numbers of marbles buried after 20 min. (E) Representative heat map showing alteration in locomotion and exploratory behavior in OFT (distance traveled in center zone vs peripheral zone). (F) Left: numbers of entries into center zone; Middle: time spent in the center zone; Right: total distance. Data are presented as the mean ± s.e.m. **p*<0.05 and ***p*<0.01 vs. saline-exposed group by two-tailed unpaired Student’s t-test.

**Figure S3.** Decreased synaptic transmission and disrupted E-I balance in ACC neurons at the early developmental stage. (A) Representative mEPSC traces in ACC pyramidal neurons at P7. (B) Summary data for mEPSCs frequency and peak amplitude in ACC pyramidal neurons obtained from saline-exposed group and VPA-exposed group (n=10 neurons, 3 mice of saline-exposed group; n=9 neurons, 3 mice of VPA-exposed group). **p*<0.05 vs. saline-exposed group by two-tailed unpaired Student’s t-test.
